# Review on Pneumococcal Infection in Children

**DOI:** 10.7759/cureus.14913

**Published:** 2021-05-09

**Authors:** Vijayakumary Thadchanamoorthy, Kavinda Dayasiri

**Affiliations:** 1 Clinical Sciences, Faculty of Health Care Sciences, Eastern University, Batticaloa, LKA; 2 Paediatrics, Base Hospital, Mahaoya, LKA

**Keywords:** streptococcal pneumonia, pneumolysin, genetic transformation, meningitis, otitis media, sinusitis, bronchitis, septic arthritis, pneumonia, osteomyelitis

## Abstract

Childhood pneumococcal infection is a growing concern among paediatricians especially, in countries where there is no routine vaccination program against *Streptococcal pneumoniae*. The disease is associated with significant morbidity and mortality in young children particularly those who are under the age of two years. Its main virulent factors include polysaccharide capsule, autolysin, pneumolysin, choline-binding Protein A, the higher chance for genetic transformation, and the presence of pilli that facilitate enhanced binding of bacteria to host cellular surfaces. More severe and invasive pneumococcal infections are seen in children with immunodeficiencies, hypofunctional spleen, malnutrition, chronic lung disease and nephrotic syndrome. The disease spectrum includes a range of manifestations from trivial upper respiratory tract infections to severe invasive pneumococcal disease (PD). The basis of diagnosis is the isolation of bacteria in the culture of body fluids including blood. Antibiotics are best guided by sensitivity patterns and the emergence of resistance is a growing concern.

## Introduction and background

Pneumococcal disease (PD) is caused by a gram-positive bacterium, *Streptococcus pneumoniae,* known as Pneumococcus. It is the most common type of community-acquired pneumonia in children. PD is the major cause of morbidity and mortality under five years in the world, according to the World Health Organization, but mortality has been high in developing countries [1]. Though it colonises 20%-40% of healthy children's nose and throat, it is a leading cause of bacterial pneumonia, meningitis, and sepsis in children at present [1,2]. It causes severe invasive disease in young children, especially under two years [3]. There are 92 different serotypes identified, but they are grouped into 46 serogroups according to their capsular polysaccharides' immunologic similarities. Of those, ten serogroups are responsible for the most severe paediatric infections globally, and 1, 3, 6, 14, 19 & 23 are being the most common [4]. The prevalence of the disease caused by the above serotypes differs over time, with population age, ethnicity, and geography. There are 13 serotypes: 1, 3, 4, 5, 6A, 6B, 7F, 9V, 14, 18C, 19A, 19F & 23F responsible for about 80%-90% of antibiotic-resistant pneumococcal strains in most parts of the world [5-8]. Before introducing the vaccine PCV7, there was a high prevalence of globally invasive pneumococcal disease (IPD). The incidence has decreased in countries where routine vaccination was introduced with PCV7 [5,9]. Simultaneously, non-vaccine pneumococcal serotypes, especially 19A, have increased serious and invasive infection incidence. Because of this reason, 13-valent pneumococcal conjugate (PCV13) was introduced, and PCV13 contains additional six serotypes (1, 3, 5, 6A, 7F & 19A) compared to PCV7. These six additional serotypes accounted for 63% of pneumococcal infections in the paediatric population currently [10].

## Review

Epidemiology

Pneumococcal infections usually occur in children under five years old and more commonly in children less than two years [5]. Children in these age groups are more vulnerable to infection due to the immature immune system and frequent infections. During the first three months of life, children are protected from infection due to passive maternal antibodies transferred via breast milk and placenta. Meningitis is generally seen between 6 and 18 months, followed by bacteremia between 6 and 36 months. Most bone infections are seen between 3 and 34 months, while pneumonia occurs between 3 months and 5 years [11]. 25% of pneumococcal pneumonia has been associated with bacteremia [12].

WHO estimates in 2005 revealed that 1.6 million children were being killed every year by the PD, with 0.7 to 1 million of them being under five years of age. High mortality was the main concern for the development of the pneumococcal vaccine [1]. A study done in California before introducing the Pneumococcal vaccine revealed a high incidence of IPD, especially among young children below two years [13]. Another study conducted by Active Bacterial Core Surveillance (ABCs) of the Centers for Disease Control and Prevention (CDC) using population-based data before and after the introduction of PCV7 revealed a reduction of disease prevalence significantly under two years [14]. And similarly, a study conducted in other centers also showed declined incidence in countries where the vaccine was introduced [15]. The introduction of a vaccine reduces age-specific mortality due to IPD and provides herd immunity in the population [16]. However, an increase may be seen in the prevalence of non-vaccine serotypes, especially antibiotic-resistant 19A, following the successful vaccination introduction. This is partly due to the extensive use of macrolide antibiotics according to the pneumococcus dynamic compartmental transmission model [17].

Aetiology and pathogenesis

Pneumococcus is alpha-haemolytic lancet-shaped, gram-positive, catalase-negative diplococci. More than 90 serotypes (about 92 serotypes) have been identified depending on the unique polysaccharide capsules [4].

The organism is usually found in the nose and throat as a commensal in 20% to 40% of healthy children and found in plenty of places where people spend a lot of time nearby, such as daycare centers and preschools [2]. The organism attaches to the nasopharyngeal cells via the interaction of bacterial surface adhesions. This normal colonization may become infectious if the organisms are carried into nasal sinuses and Eustachian tubes, where it causes otitis media and sinusitis, respectively. Pneumonia occurs when the organism is inhaled. If alveoli macrophages fail to kill the organism leading to bacteremia and further spread to meninges, joints, bones, and peritoneal cavity, causing meningitis, brain abscess, septic arthritis, and osteomyelitis [18].

Pneumococci also express several virulent factors on cell surfaces and inside the organism that mediate severe disease and capsular polysaccharides that help escape the body's defense system. The main virulent factors and their function have been listed in Table 1. It owns pneumococcal surface proteins, which resist complement-mediated opsonization and secretes IgA1 proteases that destroy secretory IgA in the body and mediates its attachment to respiratory mucosa. Table 1 shows virulent factors in pneumococci that mediate severe infection.

**Table 1:** Virulent factors in the pneumococcus [19-22].

No	Virulent factors	Productive function in the human by organism
01	Polysaccharide capsule	Prevents phagocytosis by host immune cells by inhibiting C3b opsonization of the bacterial cells
02	Pneumolysin	Causes lysis of host cells and activates complement
03	Autolysin	Activation of this protein lyses the bacteria releasing its internal content
04	Hydrogen peroxide	Apoptosis in neuronal cells during meningitis
05	Pilli	Facilitate colonization of upper respiratory tract and increase the formation of large amounts tumour necrosis factors (TNF)
06	Choline-binding protein A	Inhibits complement-mediated opsonization of pneumococci
07	Competence for genetic transformation	Plays an important role in nasal colonization fitness and virulence

There are several risk factors for the increased pneumococcal infection incidence in humans (Table 2) [23-27].

**Table 2:** Risk factors for the pneumococcal infection in humans.

	Risk factors for frequent and severe pneumococcal infections
1	Chronic medical conditions
	Congenital or acquired humoral immunodeficiency
	Impaired phagocytosis or defective clearance of organism
	Abnormal innate immune response
	Hypofunction of the spleen (sickle cell disease)
	Absent spleen (congenital or surgical removal)
	Human immunodeficiency virus (HIV)
	Nephrotic syndrome
	Chronic lung disease
	Haematological malignancies
2	Acute infections
	Overwhelming viral infection especially influenza which damages the especially respiratory epithelium
3	Cochlear implants
4	Malnutrition
5	Chronic inhalation of smoke
6	Cerebrospinal fluid leakage
7	Certain racial and ethnic groups (Native Alaskan, Black, American Indian population and Australian Aborigines)
8	Attendance in group child care
9	Crowded living conditions

Disease manifestations

Non-invasive pneumococcus infection

1. Otitis media: *Streptococcus pneumoniae* is a nasopharyngeal commensal that spreads to the middle ear through the Eustachian tube and causes infections when the host immune mechanism is disturbed. Further, persistent nasopharyngeal carriage leads to recurrent otitis media. The clinical features are ear pain, headache, fever, and purulent ear discharge [28].

2. Sinusitis or Rhino sinusitis: Sinus infections are caused by bacteria that line the nasal cavity, most possibly pneumococcus commensals, when the host immune mechanisms are weak enough to cause infection. It is usually a secondary bacterial infection preceded by viral upper respiratory tract infection or allergy. It has been shown that it is mostly secondary to drug resistance pneumococci. The clinical features include fever, headache, postnasal dribbling, nasal discharge, and sinus tenderness [29].

3. Bronchitis: Acute bronchitis is an inflammation of bronchi leading to the production of sputum. Bacterial bronchitis secondary to pneumococcus is one of the main causes of persistent and protracted cough in children [30].

Vaccination (PCV7or PCV13) would reduce noninvasive pneumococcal infection incidence by reducing the nasal carriage [28-30].

Invasive pneumococcus infection (IPD)

1. Pneumococcal meningitis: Meningitis is the most serious manifestation of pneumococcal infection secondary to bloodstream spread. After introducing the Haemophilus influenza type b vaccine, pneumococcus was the emerging infection of meninges [31]. Pneumococcal meningitis has a wide range of diseases. It starts gradually over a period of several days from non-specific upper respiratory symptoms to fulminant disease course, and it might end up in death in 24 hours of the onset of clinical manifestations. Patients present with headaches, vomiting, fever, convulsion, neck stiffness, malaise, photophobia, and altered consciousness [32]. Unlike older children, infants do not show neck stiffness. The disease carries significant morbidity and mortality despite the prompt diagnosis and early treatment and includes neurological sequelae in 25% to 56% of survivors and death in 5% to 15% of cases. Neurological complications are hearing loss, seizures, learning disabilities, and mental dysfunction [33]. 

 2. Pneumococcal bacteremia and sepsis: Pneumococcal bacteremia is common among very young children [34]. Bacteremia might occur in conjunction with meningitis, pneumonia, and septic arthritis and also occur concurrently with a localized disease such as acute otitis media or without any focal lesions. Approximately 3% to 5% of febrile children between the ages of 3 months - 36 months are at risk for asymptomatic or occult bacteremia. Of those, 85%-95% were caused by *S. pneumonia* before introducing the vaccine [35]. As symptoms in these children are non-specific, including less activity, poor feeding and irritability, fever with chills, clammy skin, confusion, difficulty in breathing, and severe body ache, diagnosis is made only by isolating bacteria from blood cultures before commencing the antibiotic therapy. Missing occult bacteremia leads to either focal or systemic infections such as meningitis and septic arthritis [36].

3. Pneumococcus pneumonia: Pneumococcus is the leading cause of bacterial pneumonia under five years worldwide [27]. 20%-40% of all the cases of pneumococcus pneumonia may be associated with bacteremia, and the case fatality rate has been lower in children than adults [37,38]. The clinical features differ from mild upper respiratory tract infection to severe respiratory distress needing mechanical ventilation. It usually produces lobar pneumonia, which might complicate pleural effusions, pleural empyema, respiratory failure, death, and meningitis [27]. They present with fever, a cough that might be dry initially and then productive with sometimes rusty coloured sputum, chest pain, headache, and difficulty breathing. Localized signs include reduced air entry, impaired resonance, and crepitations involving a lobe [39]. There is a risk of death in children with both bacteremia pneumonia depending on the serotypes, and 3, 6A, 6B, 9N, 19A, 19F, and 23F are more likely to cause death a study [40].

4. Pneumococcal bone and joint infections: About 4% of all bacterial bone infections and 20% of all joint infections are caused by* Streptococcus pneumoniae*. Bone and joint infections account for about 3%-6% of all IPD cases. Femur and humerus are the most involved bones, and infections certainly extend to vertebrae too. The frequently involved joints are the knee and hip [12]. About 50% of these infections may have septic arthritis with osteomyelitis. Nearly 50% of bone and joint infections are also associated with bacteremia, and mortality in children with septic arthritis has been lower than in adults [12].

Diagnosis

Although inflammatory markers (white blood cell, C-reactive protein, erythrocyte sedimentation rate, cerebrospinal fluid analysis urine) are high, confirmation of pneumococcal infection is by either detection of antigen or isolation of the organism from blood culture or relevant body fluids including cerebrospinal fluid, pleural fluid, urine, ascetic fluids, secretions, and sputum. A quelling test can identify specific capsular polysaccharides (cell wall C polysaccharide) in the organism [41]. A chest X-ray can help diagnose pneumonia but not the causative organisms [10].

Antibiotic treatment

In the past, most organisms were susceptible to beta-lactam antibiotics (cephalosporins, penicillin) throughout the world. Still, increasing resistance has been observed in most areas, especially in areas with high antibiotic usage. Varying proportions of strains show resistance to cephalosporin, macrolides, tetracycline, clindamycin, and fluoroquinolones. A greater proportion of isolates are, however, sensitive to vancomycin [42].

Prevention

As the increasing prevalence of pneumococcal infection is due to the replacement of common serotypes and concerns related to multidrug resistance, vaccine prevention is the sustainable option to reduce morbidity and mortality in children. There are three types of vaccines available in many countries. 

A 23-Valent pneumococcal polysaccharide vaccine

This vaccine has purified pneumococcal polysaccharide capsular antigens of the 23 most common disease-causing serotypes. It is less effective in the paediatric population because it is poorly immunogenic in infants and children under two years of age, during which the highest incidence of local and invasive pneumococcal infection, especially in serotypes 6A, 6B, 14, 19A, 19F, and 23F is seen [43,44]. This is explained as polysaccharide antigens are T lymphocyte independent and do not induce immunogenic memory. It is unsuccessful in priming for an anamnestic or booster response with subsequent re-exposure. Besides, the vaccine fails to minimize the nasopharyngeal carriage of Streptococcus pneumonia. It fails to protect mucosal infection (e.g., otitis media and sinusitis) or prevent the spread of resistant pneumococcal strains among individuals [44]. Further, the immunity declines over the first three to five years, especially in patients with asplenia and sickle cell disease [43,45]. The efficacy has been reported to be 56% to 86% in all individuals, and this vaccine reveals no protection against non-bacteremic pneumonia in all age groups [46].

Protein-conjugate vaccine (PCV7, PCV13)

It has been invented to reduce the problem of reduced immunogenicity in infants and children with polysaccharide vaccines. This vaccine is formed with epidemiologically most important pneumococcal serotypes combined with various protein carriers, including covalent linking of the polysaccharide to a protein to enhance immunogenicity. These protein carriers are T-cell-dependent antigens that stimulate a T-helper cell's response, which prime an anamnestic or booster response in individuals who are vaccinated with conjugate vaccine [44]. There are two main conjugate vaccines - PCV7 and PCV13. The PCV7 includes 4, 6B, 9V, 14, 18C, 19F and 23F [47]. The PCV13 includes six additional serotypes with PCV7 (1, 3, 5, 6A, 7F, and 19A), causing 92% of IPD and gives protection against IPD and otitis media [10,48]. This multivalent vaccine gives better protection for invasive pneumococcal infections.

A 10-valent pneumococcal conjugate vaccine (PHiD-CV)

It is a recombinant form of non-typeable Haemophilus influenza (NTHI) protein D as a protein carrier for 8 of 10 vaccine serotypes and diphtheria and tetanus toxoid as the carrier proteins for the other 2 serotypes and licensed in most of the countries since 2009 to prevent IPD, otitis media and non-typeable H. influenza by the WHO. This vaccine has 1, 5, and 7F in addition to PCV7. This vaccine is 90% effective for IPD. This vaccine can be administrated with other routine vaccinations at 2, 4, and 6 months. The vaccine also reduces vaccine-type pneumococcal nasal carriage after the primary series, and a booster dose is recommended between 12 to 15 months [49].

As there is success in developed countries following pneumococcal vaccine and also intension to reduce morbidity and mortality due to pneumococcal infection, the GAVI Alliance (in alliance with the Bill & Melinda Gates Foundation) has started to introduce the pneumococcal conjugate vaccine into the National Immunization Program of GAVI eligible countries [50].

## Conclusions

Childhood pneumococcal infection due to *Streptococcus pneumoniae* is the principal cause of morbidity and mortality worldwide. It affects children under five years, especially under the age of two years. Nonetheless, the incidence has been reduced significantly with the introduction of PCV7. The occurrence of replacement serotypes and multidrug-resistance related serotypes warrant a new introduction of PCV13 that gives added benefits of extended protection for children. There is a critical need to focus on the continued development of a vaccine with the reformulation or expansion of protein conjugate regularly every 5 to 10 years to provide immunity to all pneumococcal serotypes causing disease in humans and prevent nasopharyngeal colonization.
